# Action video gaming and the brain: fMRI effects without behavioral effects in visual and verbal cognitive tasks

**DOI:** 10.1002/brb3.877

**Published:** 2017-12-16

**Authors:** Fabio Richlan, Juliane Schubert, Rebecca Mayer, Florian Hutzler, Martin Kronbichler

**Affiliations:** ^1^ Centre for Cognitive Neuroscience University of Salzburg Salzburg Austria; ^2^ Department of Psychology University of Salzburg Salzburg Austria; ^3^ Neuroscience Institute Christian‐Doppler‐Klinik Paracelsus Medical University Salzburg Austria

**Keywords:** cognition, cognitive neuroscience, functional neuroimaging, magnetic resonance imaging, video games

## Abstract

**Introduction:**

In this functional magnetic resonance imaging (fMRI) study, we compared task performance together with brain activation in a visuospatial task (VST) and a letter detection task (LDT) between longtime action video gamers (*N *=* *14) and nongamers (*N *=* *14) in order to investigate possible effects of gaming on cognitive and brain abilities.

**Methods:**

Based on previous research, we expected advantages in performance for experienced action video gamers accompanied by less activation (due to higher efficiency) as measured by fMRI in the frontoparietal attention network.

**Results:**

Contrary to these expectations, we did not find differences in overall task performance, nor in brain activation during the VST. We identified, however, a significantly different increase in the BOLD signal from a baseline task to the LDT in action video gamers compared with nongamers. This increased activation was evident in a number of frontoparietal regions including the left middle paracingulate cortex, the left superior frontal sulcus, the opercular part of the left inferior frontal gyrus, and the left and right posterior parietal cortex. Furthermore, we found increased activation in the triangular part of the left inferior frontal gyrus in gamers relative to nongamers when activation during the LDT was compared with activation during the VST.

**Conclusions:**

In sum, the expected positive relation between action video game experience and cognitive performance could not be confirmed. Despite their comparable task performance, however, gamers and nongamers exhibited clear‐cut differences in brain activation patterns presumably reflecting differences in neural engagement, especially during verbal cognitive tasks.

## 1 INTRODUCTION

In the last years there has been increasing interest in the possible effects of video gaming on various cognitive functions. For example, Powers, Brooks, Aldrich, Palladino, and Alfieri (2013) conducted a meta‐analysis concerning the effects of video games on information processing. They combined findings from quasi‐experimental studies, in which video game players (VGP) were compared with nonvideo game players (NVGP), and training studies, in which an experimental group was compared with a control group. In the quasi‐experimental studies, there were moderate to large effects of video gaming on auditory and visual processing skills and small effects on executive functions, motor skills, and spatial imagery. Furthermore, the authors found that video game training had a substantial effect on motor skills.

There is also evidence suggesting that players of action video games (AVG) may benefit from an enhanced visuospatial working memory capacity. For example, Boot, Kramer, Simons, Fabiani, and Gratton (2008) found that action VGP outperformed NVGP in various visuospatial working memory tasks (i.e., multiple object tracking, mental rotation, and change detection). Similar, albeit weaker, effects were found after AVG training (e.g., Blacker, Curby, Klobusicky, & Chein, 2014; Boot et al., 2008; Green & Bavelier, 2006). Even more remarkable, Franceschini et al. (2013) found that dyslexic children improved their reading abilities after only 12 hr of AVG training.

These findings are exciting because they indicate that skills acquired or trained through video games could be transferred to various cognitive tasks relevant for everyday life. Several theories have been formulated to explain this broad transfer. It has been argued that AVG share a number of perceptual and attentional demands (such as multiple object tracking, rapid attentional switches, and peripheral vision) with common cognitive tasks (Oei & Patterson, 2014). In contrast, Green and Bavelier (2012) proposed that action video gaming rather unspecifically enhances the ability to learn new tasks. There is promising evidence that video game experience results in general improvement in attentional control, which, in turn, can be applied to various cognitive tasks (for a review, see Hubert‐Wallander, Green, & Bavelier, 2011). For example, Chisholm and Kingstone (2015) found that action VGP outperform NVGP in selection‐based as well as response‐based processes of an oculomotor capture task indicating that experienced gamers benefit from enhanced attentional control. Advantages for action VGP in various attention‐demanding tasks have also been reported by Cardoso‐Leite et al. (2016).

Although numerous studies investigated the effects of video gaming on the behavioral level, the neural processes underlying these effects received relatively little attention so far. Findings from studies using electroencephalography (EEG) suggest that VPG may have an advantage over NVGP in selective visual attention tasks because they more effectively suppress distracting information (e.g., Krishnan, Kang, Sperling, & Srinivasan, 2013; Mishra, Zinni, Bavelier, & Hillyard, 2011). These results are in line with the assumption that action VGP benefit from an enhanced top‐down control of attention. Wu et al. (2012) conducted an experiment where they compared event‐related potentials (ERPs) during an attentional visual field task before and after 10 hr of AVG playing. They found that the participants, who exhibited the greatest improvement in task performance, showed increased amplitudes in the visual ERPs recorded after the training. These ERPs are thought to reflect enhanced top‐down attention via active suppression of distracting information.

Recent evidence from magnetic resonance imaging (MRI) suggests that extensive video gaming might also induce changes in brain connectivity and brain structure. Gong et al. (2015) found that action VGP exhibited increased functional connectivity between attentional and sensorimotor networks as well as increased gray matter volume in insular subregions. Another study by Tanaka et al. (2013) revealed that action VGP had larger gray matter volume in the right posterior parietal cortex, which, in turn, was correlated with individual performance in a visual short‐term memory task. In a series of structural MRI studies using voxel‐based morphometry and surface‐based methods, Kühn and colleagues reported associations between video game playing and gray matter volume in the left striatum (Kühn et al., 2011), the bilateral entorhinal, hippocampal, and occipital cortex (Kühn & Gallinat, 2014), and cortical thickness in the left dorsolateral prefrontal cortex and frontal eye fields (Kühn et al., 2014). These findings were interpreted as reflecting adaptive neural plasticity in various different cognitive domains such as reward processing, navigation, visual attention, executive control, strategic planning, and visuomotor integration.

Quasi‐experimental studies comparing VGP and NVGP using functional magnetic resonance imaging (fMRI), however, are still rare in the field of AVG (see Palaus, Marron, Viejo‐Sobera, & Redolar‐Ripoll, 2017 for a comprehensive overview of brain imaging studies in the broader field of video gaming). Bavelier, Achtman, Mani, and Föcker (2012) conducted an fMRI study, in which they compared the blood–oxygen‐level‐dependent (BOLD) signals of action VGP and NVGP during a visual search task. They contrasted an easy version versus a more difficult, attention‐demanding version of the task in order to assess brain activation associated with increased attentional demands. As task difficulty was increased, all participants showed stronger activation of the frontoparietal attention network. Consistent with the hypothesis that action VGP benefit from a more efficient allocation of attention, VGP exhibited significantly weaker activation of the frontoparietal attention network compared with NVGP, while outperforming nongamers with respect to reaction times. The present study sought to build on these findings, by further comparing brain activation underlying behavioral differences between VGP and NVGP. Specifically, the aim of the present study was to investigate differences in performance as well as differences in brain activation between longtime action VGP and NVGP during a visuospatial task and a verbal letter detection task (adopted from Stephan et al., 2003).

First, we expected differences between VGP and NVGP on the behavioral level: As indicated by previous research, action VGP should show improved performance, that is, higher accuracy rates and faster reaction times in the visuospatial task compared with NVGP. Similar advantages in performance would be expected for the verbal letter detection task, if longtime action VGP indeed profit from enhanced top‐down attentional control, as proposed by Hubert‐Wallander et al. (2011).

Second, we expected differences in brain activation. If longtime AVG playing indeed enhances attentional control, we would expect to find altered activation in the bilateral dorsal frontoparietal network in action VGP compared with NVGP during both tasks, as this network is known to play a crucial role in goal‐directed top‐down control of attention (e.g., Corbetta & Shulman, 2002). In contrast, if action VGP benefit from an enhanced visuospatial working memory only, we would expect that VGP—compared with NVGP—would show altered activation in the posterior parietal cortex of the right hemisphere (a region proposed by Stephan et al., 2003) during the visuospatial task, but no difference in brain activation between the two groups during the verbal letter detection task.

## 2 MATERIAL AND METHODS

### 2.1 Participants

A total of 30 healthy university students were recruited. One participant was excluded from analysis due to severe head movements during the scanning session (exclusion criterion: movement >3 mm in any direction during a functional run) and a second participant was excluded due to technical malfunction during data acquisition. Overall, participant motion in the MRI scanner was low (median of 0.75 mm in any direction during the whole scanning session) and substantially smaller than the size of a voxel. The remaining 28 participants were aged between 18 and 29 years (*M *=* *23.04 years, *SD *= 3.07 years), had normal or corrected to normal vision, and reported no history of neurological or psychiatric disease. They received either 10 Euro or course credit for their studies and an image of their brain on DVD. All participants gave written informed consent and the aim of the study was explained to them at the end of the experiment. The study conformed to the Declaration of Helsinki and was approved by the ethical review committee of the University of Salzburg.

The amount of time spent playing AVG during the last 6 months was assessed via a German adaption of the interview by Green and Bavelier (2007). Based on these data, participants were divided into two groups: video game players (VGP) and nonvideo game players (NVGP). VGP by definition had spent at least 5 hr a week on average during the last 6 months playing AVG. Participants were defined as NVGP if they reported to have played no AVG during the last 6 months, although they could have played other types of video games. The most common types of AVG played by our VGP were first‐ and third‐person shooters. Potential limitations of this standard classification in the literature are put forward in the discussion part of this paper (section 3.1). The group of VGP (*N *=* *14) consisted of seven males and seven females with a mean age of 22.50 years (*SD *= 2.96 years) and the group of NVGP (*N *=* *14) consisted of seven males and seven females with a mean age of 23.57 years (*SD *= 3.20 years). A post hoc analysis of achieved power, given effect size *d* of 0.80 and *alpha* error probability of 0.05, resulted in statistical power of 0.53 (Faul, Erdfelder, Lang, & Buchner, 2007).

### 2.2 Behavioral tests

Handedness was assessed with a short questionnaire adapted from the Edinburgh Handedness Inventory (Oldfield, 1971). In order to ensure normal reading abilities of all participants, we used a sentence reading test currently under development in our laboratory. The test requires judging the semantic correctness of sentences within a time limit of 3 min. In addition, working memory span was assessed via the Operation Span Task (OSPAN) (Unsworth, Heitz, Schrock, & Engle, 2005). We also assessed language comprehension, logical reasoning ability, and processing speed with three subtests (vocabulary, matrix reasoning, and digit symbol coding) of the German version of the Wechsler Adult Intelligence Scale (HAWIE–R) (Tewes, 1991). Furthermore, all participants completed the Adult Self‐Report (ASR/18‐59) (Achenbach & Rescorla, 2003), taking into account only *DSM*‐oriented scales. The results of all behavioral tests are listed in Table 1. There were no significant differences between VGP and NVGP and none of the *t*‐values exceeded 1.50.

**Table 1:** Behavioral assessment of video game players (VGP) and nonvideo game players (NVGP)

	VGP (*N *=* *14)^a^		NVGP (*N *=* *14)		*t*‐tests	
	*M*	SD	*M*	SD	*t*	*p*
Sentence reading test (number of correct sentences within 3 min)	59.42	9.73	56.50	8.70	−0.81	.427
Matrix reasoning	11.50	1.83	12.00	2.32	0.60	.553
Digit symbol‐coding	11.58	2.47	11.21	2.81	−0.35	.727
Vocabulary	16.92	1.56	16.29	1.90	−0.92	.369
Operation span task	39.92	15.30	41.36	22.17	0.19	.851
ASR depressive problems	6.50	4.12	4.29	5.99	−1.08	.292
ASR anxiety problems	3.83	2.59	2.79	3.49	−0.86	.400
ASR somatic problems	0.83	1.19	2.29	3.15	1.50	.146
ASR avoidance personality problems	3.00	2.09	1.93	1.94	−1.36	.188
ASR AD/H1^b^	7.75	3.62	6.57	3.96	−0.79	.439
ASR AD/H2^b^	3.83	2.29	2.86	2.14	−1.12	.273
ASR AD/H3^b^	3.92	2.07	3.71	2.23	−0.24	.814
ASR antisocial personality problems	5.58	2.97	4.14	3.66	−1.09	.286

^a^Behavioral data are missing for two participants. ASR, “Adult Self‐Report”; ^b^Numbers of AD/H scales refer to 1, inattention and hyperactivity; 2, inattention; 3, hyperactivity and impulsivity.

### 2.3 Experimental procedure

**Figure 1:**
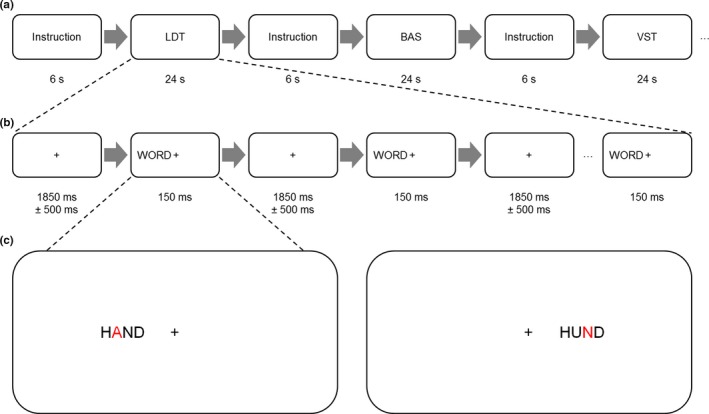
Schematic illustration of (a) the experimental procedure and (b) a task block. (c) Example of a stimulus in the left visual field containing the letter “A,” with a red letter on the second position (left) and an example of a stimulus in the right visual field not containing the letter “A,” with a red letter on the third position (right). BAS, baseline task; LDT, letter detection task; VST, visuospatial task

Before entering the scanner, the procedure and the different tasks were explained to the participants and they could ask questions if details remained unclear. In addition, all participants completed a training session (about ten minutes) outside the scanner to ensure that the tasks have been fully understood. In the scanner, participants were asked to complete a letter detection task (LDT), a visuospatial task (VST), and a baseline task (BAS) adopted from Stephan et al. (2003). The whole scanning session was subdivided into four runs, separated by 1‐min breaks, with a duration of about 10 min for each run. A run consisted of eight experimental task blocks (four LDT and four VST blocks), which were alternated with eight BAS blocks and instructions before each block. All task blocks consisted of 12 trials and lasted 24 s, while instructions lasted 6 s (for a schematic illustration of parts and blocks, see Figure 1a,b). For all three tasks, different sets of 388 words were created, which were drawn from a pool of 194 common German nouns, all comprising four letters (frequency of occurrence was controlled via the Leipzig Wortschatz library http://wortschatz.uni-leipzig.de/). In all tasks, half of the words presented contained a target letter “A” and either the second or the third letter was colored in red (for an example, see Figure 1c). Stimuli were assigned randomly to the tasks and occurrence of the target letter as well as position of the colored letter were balanced between conditions. Items were written in uppercase letters in a sans serif monospace font, which resulted in a height of 2.3° and a width of 10° visual angle. Words appeared 6° lateral to a central fixation cross in either the right or the left visual field with a stimulus onset asynchrony of 2,000 ± 500 ms (temporal jitter). Presentation field was alternated between blocks. Item presentation lasted 150 ms preventing saccades to the item to ensure that perception was only parafoveal.

In the LDT, participants were asked to indicate whether the stimulus word contained the letter “A,” irrespective of the color. Participants were instructed to press a button with their index finger for words containing the target letter and press a different button with their middle finger for words not containing the target letter. Responses were given with the right or the left hand as instructed beforehand. In the VST, participants were asked to decide whether the red letter was left or right of the word center and ignore all other features. If participants perceived the red letter on the left side of the word center, they pressed the left button, and if they perceived the red letter on the ride side of the word, they pressed the right button. Again, participants were instructed to use either their right or their left hand. For the BAS, a similar stimulus set was presented, but participants only had to press a button as quickly as possible after stimulus onset. Responses were given with the right or the left hand, with either the index finger or the middle finger as previously instructed. Condition order was pseudorandomized and counterbalanced across participants. In total, the combination of the factors task (LDT, VST, BAS), response hand (left, right), and visual field (left, right) resulted in a total of 12 conditions.

### 2.4 Data acquisition and analysis

Behavioral data were analyzed using IBM SPSS Statistics 22.0. We conducted two separate repeated measures ANOVAs for accuracy rate and reaction time as dependent variables, respectively. Group (VGP, NVGP) was set as between‐participants factor and task (LDT, VST, BAS), visual field (left, right), and response hand (left, right) were set as within‐participants factors.

A Siemens Magnetom Trio 3‐Tesla scanner equipped with a 32‐channel head coil at the Neuroscience Institute of the Christian‐Doppler‐Klinik in Salzburg was used for scanning. Functional images sensitive to BOLD contrast were acquired with a T2*‐weighted gradient echo EPI sequence (TR 2,250 ms, TE 30 ms, matrix 64 × 64, FOV 192 mm, flip angle 70°). Thirty‐six slices with a slice thickness of 3 mm and a slice gap of 0.3 mm were acquired within the TR. The scan procedure encompassed four runs with 264 scans per run. In addition to the functional images, a gradient echo field map (TR 488 ms, TE 1 = 4.49 ms, TE 2 = 6.95 ms) and a high‐resolution (1 × 1 × 1 mm) structural scan with a T1‐weighted MPRAGE sequence were acquired from each participant.

MRI data were preprocessed and analyzed using SPM8 and SPM12 software (The Welcome Department of Cognitive Neurology, London, UK, http://www.fil.ion.ucl.ac.uk/spm) running in a MATLAB 8.1 environment (Mathworks, Inc., Natick, MA, USA). Functional images were corrected for geometric distortions by the use of the FieldMap toolbox, realigned and unwarped, slice time corrected, and coregistered to the high‐resolution structural image. The structural image was normalized to the MNI T1 template image and the resulting parameters were used for normalization of the functional images, which were resampled to isotropic 3 × 3 × 3 mm voxels and smoothed with a 6‐mm FWHM Gaussian kernel.

Statistical analysis was performed in a two‐stage mixed effects model. In a participant‐specific first‐level model, the onsets of the correctly responded stimuli (hits) were modeled by a canonical hemodynamic response function with no time and dispersion derivatives. Incorrectly responded stimuli, misses, and the movement parameters derived from the realignment step during preprocessing were modeled as covariates of no interest. The functional data of these first‐level models were high‐pass filtered with a cut‐off of 128 s and corrected for autocorrelation by an AR(1) model (Friston et al., 2002). In the first‐level models, the parameter estimates reflecting signal change for each individual condition versus an implicit baseline (which consisted of the interstimulus intervals) were calculated in the context of a GLM (Henson, 2004). These participant‐specific contrast images were used for the second‐level random effects analysis. Activation for differences between tasks (LDT, VST, BAS) and groups (VGP, NVGP) were examined by *t*‐tests thresholded at a voxel level (height) of *p *<* *.001 (uncorrected) and a cluster level (extent) of *p *<* *.05 (corrected for multiple comparisons using the false discovery rate).

## 3 RESULTS

### 3.1 Behavioral results

Descriptive statistics for VGP and NVGP are shown in Table 2. Contrary to our expectations, there were no statistically significant overall differences between groups in accuracy rate, *F*(1, 26)* *= 1.36, *p *=* *.255, or reaction time, *F*(1, 26)* *= 0.52, *p *=* *.477. We found a significant hand × group interaction, indicating that VGP showed faster reaction times compared with NVGP when the left hand was used, *F*(1, 26)* *= 5.53, *p *=* *.027, η²* *= 0.18. The effect remained statistically significant when all left‐handers (*N *=* *6, 3 VGP + 3 NVGP) were excluded from analysis. There were also several other statistically significant main effects and interactions (listed in detail in Table 3), which were, however, not of primary interest in the current study.

**Table 2:** Mean (*SD*) accuracy rates and reaction times for video game players (VGP) and nonvideo game players (NVGP)

	LDT				VST				BAS			
	RH		LH		RH		LH		RH		LH	
	RVF	LVF	RVF	LVF	RVF	LVF	RVF	LVF	RVF	LVF	RVF	LVF
Accuracy rate (% correct)												
VGP	94 (4)	90 (6)	93 (5)	87 (5)	92 (6)	94 (4)	88 (9)	92 (4)	97 (4)	94 (6)	97 (4)	95 (5)
NVGP	92 (6)	88 (6)	92 (7)	87 (6)	91 (4)	90 (5)	83 (9)	90 (7)	95 (5)	95 (5)	95 (6)	94 (5)
Reaction time (ms)												
VGP	620 (60)	657 (75)	658 (55)	675 (75)	569 (109)	555 (76)	597 (88)	573 (109)	343 (34)	357 (32)	341 (40)	331 (41)
NVGP	637 (62)	680 (67)	698 895)	697 (59)	562 (77)	555 (76)	622 (104)	602 (101)	349 (49)	363 (51)	345 (44)	346 (48)

BAS, baseline task; LDT, letter detection task; LH, left hand; LVF, left visual field; RH, right hand; RVF, right visual field; VST, visuospatial task.

**Table 3:** Four‐way ANOVA results (*F*‐values, *p*‐values, and effect size) for all significant effects

	*F*	*p*	η²	Description
Reaction time				
Task	342.39	<.001	0.93	
Helmert level 1	560.17	<.001	0.96	BAS < VST + LDT
Helmert level 2	54.05	<.001	0.68	VST < LDT
Hand	38.08	<.001	0.60	RH < LH
Hand × Group	6.53	.027	0.18	For LH: VGP < NVGP
Task × Hand	19.06	<.001	0.42	For VST: RH < LH
Task × Visual field	15.52	<.001	0.37	For LDT: RVF < LVF/for VST: LVF < RVF
Hand × Visual field	9.02	.006	0.26	For RH: RVF < LVF/for LH: LVF < RVF
Accuracy rate				
Task	21.34	<.001	0.45	
Helmert level 1	33.13	<.001	0.56	BAS > VST + LDT
Hand	8.27	.008	0.24	RH > LH
Visual field	5.48	.027	0.17	RVF > LVF
Task × Hand	6.91	.002	0.21	For VST: RH > LH
Task × Visual field	30.32	<.001	0.54	For LDT: RVF > LVF/for VST: LVF > RVF

BAS, baseline task; LDT, letter detection task; LH, left hand; LVF, left visual field; RH, right hand; RVF, right visual field; VST, visuospatial task.

### 3.2 fMRI results

**Figure 2:**
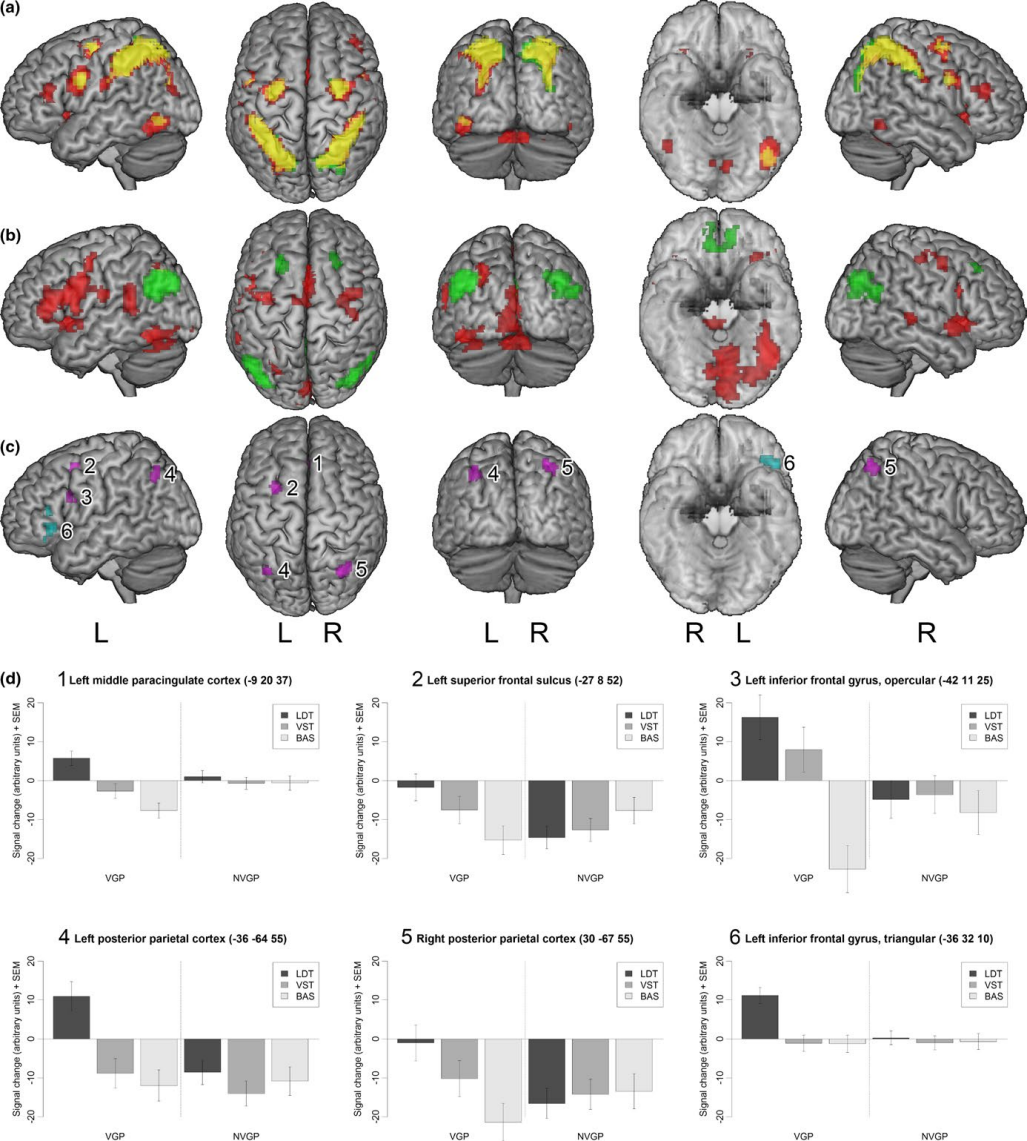
(a) Activation for the letter detection task (LDT, red) and the visuospatial task (VST, green) compared with the baseline task (BAS, overlapping regions are shown in yellow). (b) Activation for the letter detection task compared with the visuospatial task (red) and vice versa (green). (c) Activation for video gamers (VGP) compared with nonvideo gamers (NVGP) for the letter detection task compared with the baseline task (violet) and compared with the visuospatial task (blue). (d) BOLD signal change estimates for all tasks in regions with significant group differences

First, we identified brain activation during the two experimental tasks (VST, LDT) compared with the baseline task (BAS). During the VST, participants showed bilateral activation in various frontoparietal regions—from the ventral and dorsal aspects of the precentral gyrus to the supramarginal gyrus and the posterior parietal cortex together with the left occipitotemporal cortex. The same regions were found to be activated during the LDT, with additional activation in the middle frontal gyrus, the anterior insula, the occipitotemporal cortex in both hemispheres, as well as in the left and right cerebellum (a detailed overview of activated regions during both tasks is provided in Table 4). As shown in Figure 2a, activation clusters overlapped during both tasks, although a more widespread and spatially extended activation was evident during the LDT.

**Table 4:** Regions of activation for the letter detection task (LDT) and the visuospatial task (VST) relative to the baseline task (BAS)

	MNI coordinates			*Z*‐value	Extent (voxels)
	*x*	*y*	*z*		
LDT > BAS					
Left supramarginal gyrus	−39	−37	37	>8	1195
Left posterior parietal cortex	−24	−67	34	>8	
Right posterior parietal cortex	30	−61	49	>8	1059
Right supramarginal gyrus	42	−31	40	>8	
Left dorsal precentral gyrus	−27	−7	52	>8	968
Left ventral precentral gyrus	−45	2	28	>8	
Left middle paracingulate cortex	−9	14	40	>8	
Right dorsal precentral gyrus	30	−4	52	>8	292
Right ventral precentral gyrus	42	5	25	6.92	168
Left middle frontal gyrus	−36	29	13	6.50	121
Right middle frontal gyrus	39	38	19	4.73	106
Left anterior insula	−27	23	−2	6.46	104
Right anterior insula	33	23	−2	6.38	146
Left occipitotemporal cortex	−42	−61	−11	7.50	185
Right occipitotemporal cortex	48	−58	−11	4.18	24
Left cerebellum	−24	−58	−29	5.81	50
Right cerebellum	−6	−76	−23	6.92	172
VST > BAS					
Left supramarginal gyrus	−36	−37	37	>8	986
Left posterior parietal cortex	−21	−64	52	7.43	
Right posterior parietal cortex	24	−64	52	>8	1052
Right supramarginal gyrus	42	−31	40	>8	
Left dorsal precentral gyrus	−27	−7	52	>8	250
Left ventral precentral gyrus	−48	2	28	6.39	86
Right dorsal precentral gyrus	27	−4	52	7.02	196
Right ventral precentral gyrus	45	5	28	4.85	60
Left occipitotemporal cortex	−39	−64	−8	4.21	43

Second, we compared the BOLD signal directly between the two experimental tasks (see Figure 2b). Higher activation during the VST compared with the LDT was identified bilaterally in the precuneus, the anterior part of the paracingulate cortex, the superior frontal sulcus, and the angular gyrus. Conversely, higher activation during the LDT compared with the VST was found in the inferior frontal gyrus, the anterior insula, the middle paracingulate cortex, and the superior temporal gyrus of both hemispheres as well as in the left lateral prefrontal cortex, the left posterior parietal cortex, the left occipitotemporal cortex, the right dorsal precentral gyrus and the brain stem (see Table 5).

**Table 5:** Regions of activation for the letter detection task (LDT) relative to the visuospatial task (VST)

	MNI coordinates			*Z*‐value	Extent (voxels)
	*x*	*y*	*z*		
LDT > VST					
Left lateral prefrontal cortex	−39	8	22	>8	1218
Left inferior frontal gyrus, triangular	−36	29	13	7.38	
Left anterior insula	−30	23	−2	6.45	
Right dorsal precentral gyrus	45	−1	49	4.57	106
Right inferior frontal gyrus, opercular	45	14	25	4.53	69
Right anterior insula	33	26	−2	6.07	324
Bilateral middle paracingulate cortex	6	14	40	6.48	555
Left occipitotemporal cortex	−45	−58	−17	7.18	1275
Left posterior parietal cortex	−24	−67	34	6.83	219
Left superior temporal gyrus	−60	−43	16	4.96	109
Right superior temporal sulcus	51	−31	1	4.84	78
Bilateral brain stem	9	−31	−8	3.86	44
VST > LDT					
Left angular gyrus	−39	−79	31	5.77	294
Right angular gyrus	42	−76	34	5.30	203
Bilateral precuneus	−12	−58	34	5.01	555
Bilateral anterior paracingulate cortex	−9	47	−8	4.31	127
Left superior frontal sulcus	−24	23	49	4.50	80
Right superior frontal sulcus	27	23	37	4.00	56

A main objective of the current study was to directly compare brain activation between VGP and NVGP. First, there were no statistically significant differences in brain activation during the BAS. Second, and against our expectations, the BOLD signal change during the VST compared with the BAS did not differ between VGP and NVGP. Third, for the LDT compared with the BAS, we found that VGP showed higher activation in left frontal and bilateral parietal regions compared with NVGP. Specifically, this difference in activation was present in the left middle paracingulate cortex, the left superior frontal sulcus, the opercular part of the left inferior frontal gyrus, and the left and right posterior parietal cortex (see Table 6). Fourth, we searched for differences in brain activation between VGP and NVGP, comparing the LDT with the VST. We found that VGP exhibited higher activation than NVGP during the LDT relative to the VST. This difference was evident in the triangular part of the left inferior frontal gyrus (see Table 6). All regions with higher activation in VGP compared with NVGP are shown in Figure 2c. We did not find any regions with higher activation in NVGP compared with VGP, neither for the experimental tasks compared with the BAS nor for the experimental tasks compared with each other.

**Table 6:** Regions of activation for the letter detection task (LDT) relative to the baseline task (BAS) and the visuospatial task (VST) in gamers (VGP) compared with nongamers (NVGP)

	MNI coordinates			*Z*‐value	Extent (voxels)
	*x*	*y*	*z*		
VGP > NVGP: LDT > BAS					
Left middle paracingulate cortex	−9	20	37	4.93	106
Left superior frontal sulcus	−27	8	52	4.60	
Left inferior frontal gyrus, opercular	−42	11	25	4.38	40
Left posterior parietal cortex	−36	−64	46	3.90	59
Right posterior parietal cortex	30	−67	55	4.71	41
VGP > NVGP: LDT > VST					
Left inferior frontal gyrus, triangular	−36	32	10	5.07	90

Figure 2d illustrates the BOLD signal change estimates for the regions identified with group differences. In the left middle paracingulate cortex, VGP displayed an increase in the BOLD signal during the LDT, which was significantly different from the decrease in the signal during the BAS, whereas NVGP did not show any change in activation in this region. In VGP, the left superior frontal sulcus was deactivated during the BAS, but this decrease in activation was no longer visible during the LDT. In NVGP, however, the same region was deactivated during all three tasks. In the opercular part of the left inferior frontal gyrus, VGP showed an increased BOLD signal during the LDT and a decreased signal during the BAS, whereas NVGP, once again, showed a decrease in BOLD signal during all three tasks. Similarly, in the left posterior parietal cortex, VGP showed signal increase during the LDT and decrease during the BAS, whereas NVGP showed signal decrease during all three tasks. A slightly different pattern was evident for the right posterior parietal cortex. Here, VGP did not exhibit any BOLD signal change during the LDT, but displayed a decrease during the BAS. In contrast, in NVGP, this decrease was found during all three tasks. In the triangular part of the left inferior frontal gyrus, VGP displayed an increase in BOLD signal during the LDT and a decrease during the VST, whereas NVGP showed a decrease during all tasks. Additionally, we found an increase from BAS to LDT in VGP, which, however, did not survive the cluster extent threshold of *p *<* *0.05 FDR‐corrected in the whole‐brain analysis, because the cluster consisted of only 18 voxels.

**Figure 3:**
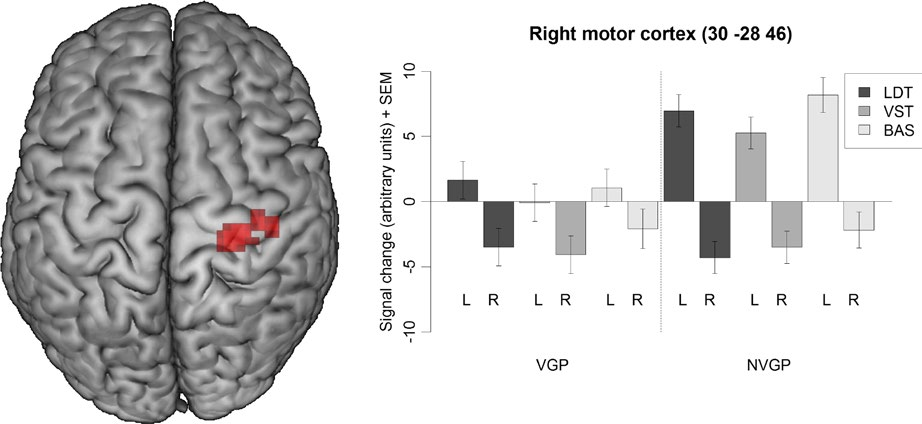
Activation for responses with the left (L) hand relative to responses with the right (R) hand in gamers (VGP) compared with nongamers (NVGP). BAS, baseline task; LDT, letter detection task; VST, visuospatial task

Since on the behavioral level the only difference between VGP and NVGP was found with respect to whether the responses were given with the left or the right hand, we conducted additional fMRI analyses targeted at this effect. To recapitulate, VGP compared with NVGP showed faster reaction times when responses were required to be given with the left hand. This effect was independent of the task. Regarding brain activation, we identified less activation for VGP compared with NVGP for responses with the left hand relative to the right hand in the right motor cortex and in the left cerebellum (see Table 7 and Figure 3). In line with the behavioral finding, this effect was independent of the task. No clusters were identified with higher activation for VGP compared with NVGP or with group differences for responses with the right hand relative to the left hand.

**Table 7:** Regions of activation for responses with the left hand (LH) relative to responses with the right hand (RH) in gamers (VGP) compared with nongamers (NVGP)

	MNI coordinates			*Z*‐value	Extent (voxels)
	*x*	*y*	*z*		
NVGP > VGP: LH > RH					
Right motor cortex	30	−28	46	6.43	124
Left cerebellum	−3	−58	−5	5.40	120

## 4 DISCUSSION

We investigated possible effects of longtime action video gaming on cognitive functions and neural processes underlying these functions. For this purpose, we compared VGP with NVGP during a visuospatial task and a verbal letter detection task with respect to behavioral performance and brain activation as measured by fMRI. Against our expectations, there were no statistically significant differences in performance between VGP and NVGP. That is, longtime action video gaming had no influence on accuracy rate or reaction time in our VST or LDT.

We found, however, that VGP had an advantage over NVGP, as they showed overall faster reaction times, when they were instructed to respond with their left hand. This effect was independent of the task and the participants’ handedness and was also reflected in a reduced BOLD response in the right motor cortex—presumably reflecting higher neural efficiency in VGP compared with NVGP. Typical office computer work (besides typing) and nonaction video games are usually executed primarily with the right hand operating the computer mouse. In AVG, the situation is different, as the left hand is more important. Here, it is usually the case that the right hand is moving the computer mouse (for looking or aiming), while the left hand is pressing buttons on the keyboard (for moving and jumping). This additional training of the left hand might be the underlying cause for the left hand advantage of the VGP over the NVGP across tasks. As already mentioned, one exception is typing: Here, both hands have to operate in precise spatial and temporal coordination. Typing, however, is a highly specialized and automatized skill, with little conscious access to implicit memory. Therefore, we speculate that the requirements of the present cognitive tasks (responding as fast as possible with either the index or middle finger) are more related to the motor skills trained with AVG than to the specific skills trained with typing.

Our main interest, however, was concerned with the brain activation patterns elicited by the visual and verbal cognitive tasks. We did not find any differences between VGP and NVGP regarding change in activation from the BAS to the VST. This is contrary to the assumption that gamers profit from an enhanced visuospatial working memory. We found, however, that VGP compared with NVGP showed a higher increase in activation from the BAS to the LDT. This increase in activation was evident in the left frontal lobe as well as in the left and right posterior parietal cortex. Additionally, when the BOLD signal during the LDT was compared with the signal during the VST, VGP compared with NVGP showed enhanced activation in the left inferior frontal gyrus.

Since we did not find any differences between VGP and NVGP on the behavioral level, one might conclude that longtime action video gaming had no influence on cognitive performance. There have been various studies, which also found no beneficial effects of AVG experience on cognitive performance (e.g., Collins & Freeman, 2014; Van Ravenzwaaij, Boekel, Forstmann, Ratcliff, & Wagenmakers, 2014) and we suppose that a great number of similar studies have gone unreported due to publication bias. On the other hand, there have also been many studies reporting cognitive advantages for action video gamers—as mentioned in the introduction part of this paper (section Introduction). Based on previous findings, we would have expected faster reaction times and higher accuracy rates in VGP at least for the VST, since it has been found that gamers might profit from an enhanced visuospatial working memory (e.g., Boot et al., 2008).

It should be noted that stimulus presentation in our VST was rather uncharacteristic for a visuospatial task, as the nature of the stimulus did not differ between our LDT and VST (for details, see section 2.1). This seems important, since it has been argued that common cognitive tasks, which have previously been used to investigate possible effects of AVG, share many perceptual and cognitive elements with AVG (Oei & Patterson, 2014). Our VST, in contrast, did not contain such typical elements (e.g., multiple object tracking, mental rotation, etc.) and was therefore different from typical gaming stimuli. Thus, VGP might not have been able to adjust their usual cognitive strategies to the novel perceptual demands in our VST. Following this implication, our results are at odds with Green and Bavelier's (2012) proposal that AVG playing improves the ability to learn new tasks and that cognitive and attentional benefits from AVG training generalize to various situations. Instead, the present findings indicate that previously observed effects of AVG training could, in part, be attributed to the perceptual nature of common visuospatial tasks. We suggest that future studies use a multifaceted set of cognitive tasks to investigate the hypothesis that gaming skills can be transferred to a broad number of cognitive tasks.

On the neural level, the results were also not as originally expected. Rather than a decrease in brain activation during the VST, we found an increase in activation during the LDT in VGP compared with NVGP in frontoparietal regions. Due to the fact that there were no differences between VGP and NVGP on the behavioral level, we can rule out the possibility that the difference in brain activation is a mere direct consequence of differences in task performance. Instead, we speculate that many of the brain activation differences reported in previous studies may be explained by differences in accuracy rates or reaction times, which were not accounted for in the analyses. We suggest that the here observed neural differences reflect distinct cognitive mechanisms in VGP and NVGP during our tasks. There are, however, various ways to explain the observation that VGP compared with NVGP exhibited increased frontoparietal activation from the BAS to the LDT, which will be discussed in the following section.

It seems reasonable to assume that the LDT was more demanding for VGP than for NVGP, as they exhibited higher brain activation for a comparable performance level. Higher activation was found in the left inferior frontal gyrus (IFG), a region which has also been associated with the LDT by Stephan et al. (2003). The left IFG is known to play a crucial role in semantic as well as phonological processing (Poldrack et al., 1999), and it has been found to be activated during inner speech and auditory verbal imagery (McGuire et al., 1996). Especially the opercular and triangular parts of the left IFG have been attributed to phonological processing (Burton, 2001). Considering a proposal by Perfetti and Bell (1991) that automatic phonemic activation occurs even prior to word identification, it makes sense that these regions were found to be activated during the LDT compared with the BAS. This activation difference was not observed in NVGP.

Furthermore, VGP exhibited increased activation from the BAS to the LDT in the bilateral posterior parietal cortex. Here, NVGP showed a comparably decreased signal during all three tasks. VGP, on the other hand, showed task‐dependent changes in activation. During the LDT they displayed an increased BOLD signal in the left posterior parietal cortex—more precisely in the left angular gyrus. In contrast, in the right posterior parietal cortex—around the intraparietal sulcus—this increase in activation was absent. During the VST and BAS, however, activation in the posterior parietal cortex was decreased in both hemispheres. The observation that some regions exhibit a decrease in activation during a cognitive task is not unusual. Since the discovery of the default mode network, we know that during the resting state, when the mind is free to indulge in associative processes and daydreaming, our brain is highly active (Raichle et al., 2001). The angular gyrus (bilaterally) is considered to be part of the default mode network and has been found to decrease activation (compared with a resting baseline) during various cognitive tasks (Binder, 2012). Thus, the observation that NVGP displayed a decreased signal in the posterior parietal cortex during all three tasks is in line with previous findings. Interestingly, in VGP this region was activated (in the left hemisphere) or at least not deactivated (in the right hemisphere) during the LDT. The posterior parietal cortex has been found to play an active role in episodic memory (Cabeza, Ciaramelli, Olson, & Moscovitch, 2008) and semantic knowledge retrieval (Binder, 2012). Cabeza et al. (2008) developed a model, in which they distinguish between dorsal and ventral parietal regions and between top‐down and bottom‐up attention on memory. For the ventral parietal cortex including the angular gyrus, they propose a dual function of bottom‐up attentional processes (see also Corbetta & Shulman, 2002) as well as episodic memory retrieval. According to Cabeza et al. (2008), an example of bottom‐up attention that is driven by episodic memory is involuntary remembering of events that enter consciousness and take over attentional resources. Activation related to this memory‐guided bottom‐up attention was postulated to be more pronounced in the left hemisphere. Thus, the observed activation of the left angular gyrus in VGP during the LDT could be the result of unintentional memory retrieval processes, whereas NVGP more successfully inhibited these processes. If this was the case, it had, however, no observable effect on behavioral performance, since VGP and NVGP showed comparable accuracy rates and reaction times in the LDT.

Additional regions, where we observed increased activation from BAS to LDT in VGP, but not in NVGP, were the left middle paracingulate cortex and the left superior frontal sulcus. The former is located proximal to the anterior cingulate cortex (ACC), an area which has frequently been associated with attention and cognitive control (e.g., Bush, Luu, & Posner, 2000) and found to be activated during the LDT by Stephan et al. (2003) as well. The latter (the superior frontal sulcus) has been related to working memory in the spatial domain (Courtney, Petit, Maisog, Ungerleider, & Haxby, 1998; Du Boisgueheneuc et al., 2006). Surprisingly, this region was deactivated during the VST in VGP as well as in NVGP. That is, activation in the left superior frontal sulcus was higher in the absence of a task than during the VST. Furthermore, in VGP, deactivation in this region decreased during LDT compared with VST, whereas in NVGP deactivation increased. Interpreting this finding would be highly speculative and therefore additional research is needed to clarify its implications. What is evident from the current findings, though, is that basic cognitive processes are reflected in different brain activation patterns in VGP and NVGP, despite there are no observable differences on the behavioral level.

### 4.1 Limitations and outlook for future studies

Some limitations should be considered when interpreting the results of the present study. First, one might argue that the difficulty level of our experimental tasks was not high enough to result in significant differences in performance. A closer look at the behavioral results reveals that all participants (VGP as well as NVGP) showed excellent performance in the LDT (with mean accuracy rates ranging from 87% to 94%) as well as in the VST (with mean accuracy rates ranging from 83% to 94%; see also Table 2). Since task performance is more or less at ceiling, the poor discriminating power of our tasks might have diluted possible effects. Whether benefits from longtime AVG experience would become apparent with more demanding tasks remains a question for future studies.

Second, for classification of VGP and NVGP, we used the standard criterion of at least 5 hr a week on average during the last 6 months playing AVG (Green & Bavelier, 2007). The most common types of AVG played by our VGP were first‐ and third‐person shooters. NVGP, on the other hand, reported to have played either no AVG or no video games at all during the last 6 months. Recently, Hartanto, Toh, and Yang (2016) found that the age of active onset of video game playing better predicted performance in a cognitive control task than did recent playing. In sum, future studies should emphasize a more fine‐grained classification by taking into account different subgroups of VGP and NVGP as well as recent and previous video game playing (especially during periods of high cognitive plasticity).

Third, it should be noted that we did not acquire basic differences in passive resting brain activation between VGP and NVGP. Changes in BOLD signal related to neural activation are rather small compared with the variation induced by anatomy (Buckner, 2012). The common way to bypass this issue is to compare activation during the task of interest with an active (another task) or a passive (resting) control condition. Comparing BOLD signal change differences between two groups in response to a particular task relative to a baseline task, however, could be confounded in a situation where the two groups differ with respect to their BOLD signal change variations during rest. This would make an interpretation in terms of task‐related activation or deactivation difficult. Since anatomical differences between gamers and nongamers have already been reported (e.g., Tanaka et al., 2013), it is likely that these groups also differ with respect to resting state brain activation reflecting a divergence in the recruitment of brain regions for cognitive processes. Although it is not reasonable to compare absolute BOLD signal values between groups, it has become a useful approach to compare functional connectivity during the resting state. For example, a recent study by Han, Kim, Bae, Renshaw, and Anderson (2015) found that adolescents with Internet gaming disorder displayed increased functional connectivity of the default mode and executive control networks. It might well be that differences in resting‐state functional network connectivity contribute to the observed differences in activation during the LDT. We suggest that future studies focus on the relationship between functional connectivity at rest and during tasks in action video gamers and nongamers. A similar approach has already been used in other domains, for example, comparing typical and impaired readers (Schurz et al., 2015).

### 4.2 Conclusions

We compared task performance and brain activation in a visuospatial task (VST) and a letter detection task (LDT) between action video gamers (VGP) and nongamers (NVGP) in order to investigate the effects of longtime gaming on cognitive and brain functions. There were no statistically significant differences in performance between VGP and NVGP. Regarding brain activation, we found a significantly different increase in the BOLD signal from a baseline task to the LDT in VGP compared with NVGP in the left middle paracingulate cortex, the left superior frontal sulcus, the opercular part of the left inferior frontal gyrus, and the left and right posterior parietal cortex. Furthermore, we identified increased activation in the triangular part of the left inferior frontal gyrus in VGP relative to NVGP when activation during the LDT was compared with activation during the VST. In sum, longtime action video gaming had no influence on accuracy rate or reaction time in our VST or LDT. Despite their comparable task performance, however, VGP and NVGP exhibited clear‐cut differences in brain activation patterns presumably reflecting differences in neural engagement, especially during verbal cognitive tasks.

## CONFLICT OF INTEREST

None declared.
